# Clinical phenotypes of delirium in patients admitted to the cardiac intensive care unit

**DOI:** 10.1371/journal.pone.0273965

**Published:** 2022-09-02

**Authors:** Ryoung-Eun Ko, Sungeun Kim, Jihye Lee, Sojin Park, Daehwan Bae, Ki Hong Choi, Taek Kyu Park, Chi Ryang Chung, Jeong Hoon Yang

**Affiliations:** 1 Department of Critical Care Medicine, Samsung Medical Center, Sungkyunkwan University School of Medicine, Seoul, Republic of Korea; 2 Department of Pharmaceutical Services, Samsung Medical Center, Seoul, Republic of Korea; 3 Division of Cardiology, Department of Medicine, Samsung Medical Center, Sungkyunkwan University School of Medicine, Seoul, Republic of Korea; University of Tampere, FINLAND

## Abstract

**Background:**

Limited data are available on clinical phenotype for delirium that occurs frequently among patients admitted to the cardiac intensive care unit (CICU). The objective of this study was to investigate the clinical pictures of delirium, and their association with clinical outcomes in CICU patients.

**Methods:**

A total of 4,261 patients who were admitted to the CICU between September 1 2012 to December 31 2018 were retrospectively registered. Patients were excluded if they were admitted to the CICU for less than 24 hours or had missed data. Ultimately, 2,783 patients were included in the analysis. A day of delirium was defined as any day during which at least one CAM-ICU assessment was positive. The clinical risk factors of delirium were classified by the delirium phenotype, as follows; hypoxic, septic, sedative-associated, and metabolic delirium.

**Results:**

The incidence of delirium was 24.4% at the index hospitalization in all CICU patients, and 22.6% within 7 days after CICU admission. The most common delirium phenotype was septic delirium (17.2%), followed by hypoxic delirium (16.8%). Multiple phenotypes were observed during most delirium days. Delirium most frequently occurred in patients with heart failure. Of all patients affected by delirium within 7 days, both ICU and hospital mortality significantly increased according to the combined number of delirium phenotypes.

**Conclusions:**

Delirium occurred in a quarter of patients admitted to the modern CICU and was associated with increased in-hospital mortality. Therefore, more efforts are needed to reduce the clinical risk factors of delirium, and to prevent it in order to improve clinical outcomes in the CICU.

## Introduction

The role of the Cardiac Intensive Care Unit (CICU) has been transformed by the changing demographics of cardiac patients. At the beginning of the 20^th^ century, valvular heart disease and heart failure were the main medical issues in the CICU. Since then, the management of cardiac arrest and interventions for acute myocardial infarction have grown in importance. As technology evolves, CICU practices also evolve. CICU procedures have become more invasive, including complex coronary interventions and percutaneous extracorporeal life support, and various other complex interventions. In addition, the severity of illness and comorbidities in cardiac patients have also significantly increased over time [1]. Therefore, patient candidate for admission significantly overlap between the medical intensive care unit (ICU) and CICU. The importance of general intensive care, such as acute respiratory failure, acute kidney injury, bleeding and sepsis, as well as cardiac critical care, have been emphasized more recently in the CICU.

Delirium is acute brain dysfunction that is characterized by disturbances of awareness, attention, and cognition with a fluctuating course. Delirium is linked to an underlying medical condition, and occurs frequently among patients admitted to ICUs [2]. Previous studies have found that the incidence of patients affected by delirium ranges from 20% to 80%. It mostly occurs in mechanically ventilated patients [3]. Critically ill patients that are affected by delirium are at a higher risk of dying in the hospital and ICU, having long-term cognitive impairment, and increased medical costs than are those without delirium [4, 5]. Several studies have found that the presence of delirium increases the risk of additional issues in the CICU from 8% to 20% [6, 7].

However, the clinical risk factors associated with delirium have not yet been fully evaluated in the CICU setting. Therefore, we investigated the clinical pictures of delirium, (ie, defined as phenotypes) and their association with the clinical outcomes of patients admitted to the CICU.

## Methods

### Study population

All consecutive patients who were admitted to a CICU were retrospectively registered at our institution. Between September 1 2012 and December 31 2018, a total of 4,261 patients who were 18 years old or older were enrolled. Of these patients, 1,475 were excluded because they were admitted to the CICU for less than 24 hours. An additional three patients were excluded because their data were not available. Ultimately, a total of 2,783 patients were included for the analysis. The Institutional Review Board of Samsung Medical Center approved this study and waived the requirement for informed consent because of its observational nature (IRB No. 2020-10-102). In addition, the patients’ information was anonymized and de-identified prior to analysis.

### Standard care in the cardiac intensive care unit

Our CICU is a 12-bed ICU with a 1:2 nurse-to-patient ratio. We offer level 1 care for CICU patients [8]. In order to provide comprehensive critical care to patients with all kinds of cardiovascular disease and complex comorbidities, the CICU is equipped with invasive and noninvasive devices that can monitor patients’ hemodynamic status and provide advanced therapeutic technologies to support the cardiovascular system. These devices include the intra-aortic balloon pump, extracorporeal membrane oxygenation, ventricular assist devices, mechanical ventilators, and continuous renal replacement therapy. A high-intensity staffing model was adapted in our CICU. The patients are managed at all times by a dedicated cardiac intensivist who is board certified in interventional cardiology and critical care medicine [9]. Cardiac surgery support is readily accessible. In addition, multidisciplinary care is provided through consultation with a dietitian, pharmacist, and a respiratory care practitioner.

For general intensive care, the clinical practice guidelines published by The Society of Critical Care Medicine were applied [10, 11]. The Confusion Assessment Method for the ICU (CAM-ICU) assessment was performed by nurses three times a day in patients with a Richmond Agitation-Sedation Scale (RASS) of -3 (indicating movement or eye opening to voice but no eye contact) or higher. The validated Korean version of the CAM-ICU was routinely used [12]. The recorded CAM-ICU results were re-checked every day by a senior nurse. We also used a specific protocol-based weaning program with spontaneous breathing trials for patients on mechanical ventilation [13].

### Definition and data collection

A day of delirium was defined by any day during which at least one CAM-ICU assessment was positive. To accurately evaluate the total number of days at risk of delirium, we did not include days when the patients were in a coma [14]. A coma day was defined by a RASS assessment of -4 (unresponsive to voice but responded to physical stimulation) or -5 (unresponsive to voice and physical stimulation).

To evaluate the clinical risk factors associated with delirium, we adopted the concept of delirium phenotypes as described by Girard et al. [15] The definitions of each phenotype are presented in **S1 Table**. In brief, we defined a set of four clinical delirium phenotypes, including hypoxic, septic, sedative-associated, and metabolic delirium. We reasoned that it was possible for simultaneous delirium phenotypes to coexist on any given day. In other words, patients could be classified as having more than one delirium phenotype on a delirium day. If multiple delirium phenotypes were present simultaneously, then the sum total duration of every phenotype would exceed the total number of days that the patient spent delirious. If a patient was delirious but did not meet any of the delirium phenotype definitions, then it was called ‘unclassified delirium’.

For comparing according to motor subtypes of delirium, delirium was classified for CAM-ICU positive results using the corresponding RASS score. Hypoactive delirium was defined if the RASS score was from -3 to 0, hyperactive if the RASS score was defined if the RASS score was from -3 to 0, hyperactive if the RASS score was from +1 to +4, and mixed-type delirium if both hypoactive and hyperactive delirium episodes were presented the observation period [16].

The vital signs and ventilator settings were automatically recorded hourly. Clinical information, including drug administration, laboratory test results, and clinical measurements, were recorded as real-time in electrical medical records. The data from electrical medical records are transferred daily to the Data Analytics and Research Window for Integrated knowledge (DARWIN)-C, the clinical data warehouse of Samsung Medical Center. In this study, the data was extracted from a retrospective CICU registry integrated into the DARWIN-C. The data on phenotype from patients with a positive CAM-ICU assessment were reviewed by three intensivists (R-E Ko, S Kim, and J Lee).

### Statistical analysis

Data are presented as medians and interquartile ranges (IQR) for continuous variables, and as numbers (percentages) for categorical variables. The categorical variables were analyzed by Pearson’s chi-square or Fisher’s exact tests. If there was a difference, post hoc analysis was performed. The mortality rate trend according to a combination of multiple delirium phenotypes was calculated using Poisson regression models. All of the data analyses and visualizations were performed using R Statistical Software. (Version 3.2.5; R Foundation for Statistical Computing, Vienna, Austria).

## Results

### Clinical characteristics

The clinical characteristics are shown in **Table 1**. The median age was 67.0 (IQR 56.0–76.0) years, and 64.6% of the patients were male. The most common comorbidity was hypertension (55.5%), followed by diabetes mellitus (30.5%). Three-hundred and one patients (10.8%) had a history of stroke. Forty-one patients (1.5%) were diagnosed with dementia. Most patients were admitted from the emergency room (59.1%) and admitted for scheduled and unscheduled procedures (38.0%). The most common primary diagnosis was acute coronary syndrome (43.7%), followed by acute heart failure (30.4%). The Initial Sequential Organ Failure Assessment (SOFA) score was 3.0 (IQR 2.0–6.0). A total of 275 patients (9.9%) received cardiopulmonary resuscitation at the time of CICU admission. Of all patients, 447 patients (16.1%) received vasoactive drugs, 381 patients (13.7%) were treated with mechanical ventilation, and 126 patients (4.5%) underwent extracorporeal membrane oxygenation. The median Troponin I was elevated to 1.2 (IQR 0.1–12.4) ng/mL. Overall, 115 (4.1%) patients expired in the CICU, while 188 (6.8%) patients expired at the index hospitalization. The median CICU length of stay was 2.1 (IQR 1.6–3.9) days. The characteristics and incidence of delirium in these patients are compared with those of other registries in **Table 2** [6, 7, 17].

**Table 1:** Clinical characteristics of the study population.

	Total (N = 2,783)
Age, year	67.0 (56.0–76.0)
Sex, male	1,799 (64.6)
Body mass index, kg/m^2^	23.7 (21.8–25.7)
Underlying disease	
Hypertension	1,544 (55.5)
Diabetes	848 (30.5)
Chronic lung disease	180 (6.5)
End stage renal disease on hemodialysis	115 (4.1)
Liver disease	74 (2.7)
Solid cancer	341 (12.3)
Hematologic malignancy	10 (0.4)
Stroke	301 (10.8)
Cerebral hemorrhage	76 (2.7)
Dementia	41 (1.5)
Psychotic disorder	5 (0.2)
Brain tumor	6 (0.2)
Chronic neurodegenerative disease	46 (1.7)
Admission route	
Emergency room	1,645 (59.1)
General ward	403 (14.5)
High dependency unit	4 (0.1)
Other intensive care unit	288 (10.3)
Outpatient clinic	186 (6.7)
Other hospital	256 (9.2)
Admission cause	
Medical	1,724 (61.9)
Scheduled procedure	699 (25.1)
Unscheduled procedure	359 (12.9)
Primary diagnosis	
Acute coronary syndrome	1,217 (43.7)
Heart failure	846 (30.4)
Arrhythmia	313 (11.2)
Aortic disease	180 (6.5)
Pulmonary hypertension	123 (4.4)
Pericardial disease	76 (2.7)
Other	28 (1.0)
Initial SOFA	3.0 (2.0–6.0)
Cardiac arrest at admission	275 (9.9)
Vital signs at admission	
Glasgow coma scale	15.0 (15.0–15.0)
Systolic blood pressure, mm Hg	122 (103–142)
Diastolic blood pressure, mm Hg	72.0 (60.0–84.0)
Heart rate, /min	85.0 (70.0–101.0)
Respiratory rate, /min	19.0 (16.0–22.0)
Body temperature	36.7 (36.3–37.1)
Saturation, %	98.0 (96.0–99.0)
Organ support at admission	
Vasoactive drug	447 (16.1)
Mechanical ventilation support	381 (13.7)
Intra-aortic balloon pump	29 (1.0)
Extracorporeal membrane oxygenation	126 (4.5)
Laboratory test	
White blood cell, max, 10*3/L	10.0 (7.5–13.6)
Hemoglobin, g/dL	11.8 (9.9–13.6)
Platelet, 10*3/L	180 (132–226)
Total bilirubin, mg/dL	0.9 (0.6–1.4)
Aspartate transaminase, U/L	40.0 (26.0–96.0)
Alanine transaminase, U/L	28.0 (18.0–54.0)
Albumin, g/dL	3.7 (3.2–4.1)
Blood urea nitrogen, mg/dL	21.1 (15.1–33.1)
Creatinine, mg/dL	1.1 (0.8–1.7)
C-reactive protein, mg/dL	0.9 (0.2–4.0)
Troponin I, ng/mL	1.2 (0.1–12.4)
Delirium occurrence in CICU	
During CICU stay	678 (24.4)
Within 7 days	629 (22.6)
Outcomes	
CICU length of stay	2.1 (1.6–3.9)
CICU mortality	115 (4.1)
Hospital mortality	188 (6.8)

Presented values are medians with interquartile ranges in parentheses or numbers with percentages in parentheses.

**Table 2:** Characteristics of patients in present study compared with other CICU delirium studies.

Variables	Present study	Naksuk et al. [6]	Falsini et al. [17]	Pauley et al. [7]
Population				
Number of patients	2,783	11,079	726	590
Region	Korea	USA	Italy	USA
Setting	Single center	Single center	Two centers	Single center
Time period	2012–2018	2004–2013	2014–2015	2012–2014
Exclusion	Admitted less than 24 h	Antipsychotic users	Intubated patients	Surgical
Measurement				
Method	CAM-ICU	CAM-ICU	CAM-ICU	CAM-ICU
Interval	Every 8 hours	From 8 AM to 8 PM	Once daily	Every 2 hours for MV/ every 4 hours for others
Demographics				
Age, years	67 (56–76)	67±15	79.1±7.8	64 (54–73)
Sex, male	1,779 (64.6)	6,911 (62.4)	413 (56.9)	362 (61.4)
Primary diagnosis				
Acute coronary syndrome	1,217 (43.7)	5,440 (49.1)	362 (49.9)	158 (26.8)
Heart failure	846 (30.4)	4,336 (39.1)	93 (12.8)	69 (11.7)
Cardiac arrest at admission	275 (9.9)	909 (8.2)	27 (3.7)	31 (5.3)
Clinical features				
Inotrope	447 (16.1)	2,458 (22.2)	14 (1.9)	101 (17.1)
MV support	381 (13.7)	1,687 (15.2)	None	119 (20.2)
IABP	29 (1.0)	902 (8.1)	N/A	31 (5.3)
ECMO	126 (4.5)	N/A	N/A	6 (1.0)
Outcomes				
Hospital mortality	188 (6.8)	905 (8.2)	35 (4.8)	62 (10.5)
Delirium incidence	678 (24.4)	925 (8.3)	111 (15.3)	120 (20.3)

Presented values are means ± standard deviations, medians with interquartile ranges or numbers with percentages.

CAM-ICU, Confusion Assessment Method for the intensive care unit; MV, mechanical ventilation; IABP, intra-aortic balloon pump; ECMO, extracorporeal membrane oxygenation; SOFA, Sequential Organ Failure Assessment; CICU, cardiac intensive care unit.

### Delirium in CICU

**Fig 1:**
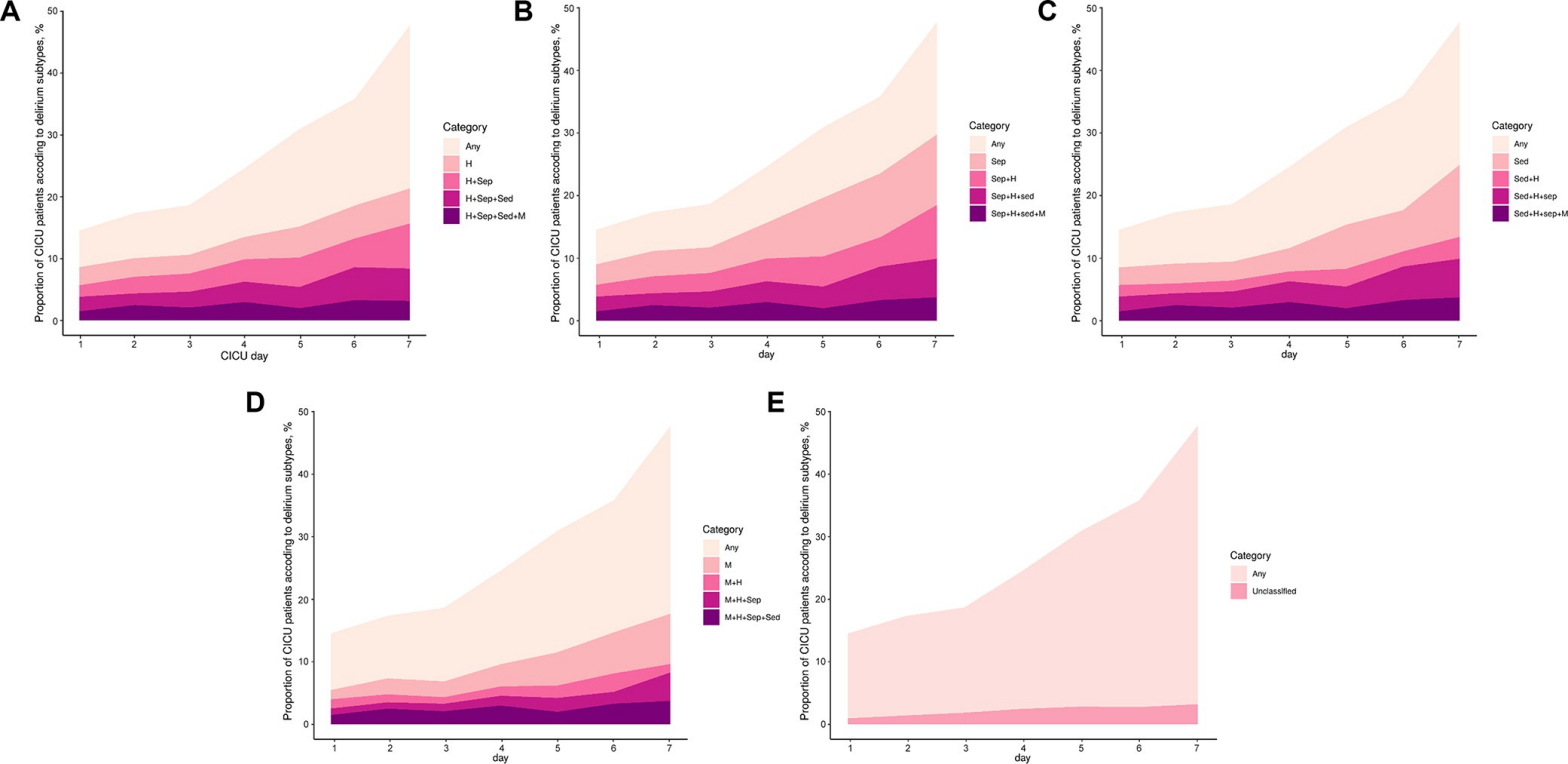
Prevalence of delirium phenotypes according to CICU admission day. (A) Hypoxic delirium, (B) Septic delirium, (C) Sedative delirium, (D) Metabolic delirium, (E) Unclassified delirium. Each area plot shows (on the y-axis) the percentage of patients in the CICU who had any delirium within 7 days, a single delirium phenotype, or a combination of delirium phenotypes according to CICU admission day (shown on the x-axis). The yellow shading indicates the overall percentage of participants with delirium on each study day. The purple lines and shaded areas represent the number of phenotypes of delirium present. Darker purples represent the presence of more delirium phenotypes. The lightest red regions show the percentage of patients with a given single phenotype. H, Hypoxic; Sep, septic; Sed, sedative; M, metabolic.

**Fig 2:**
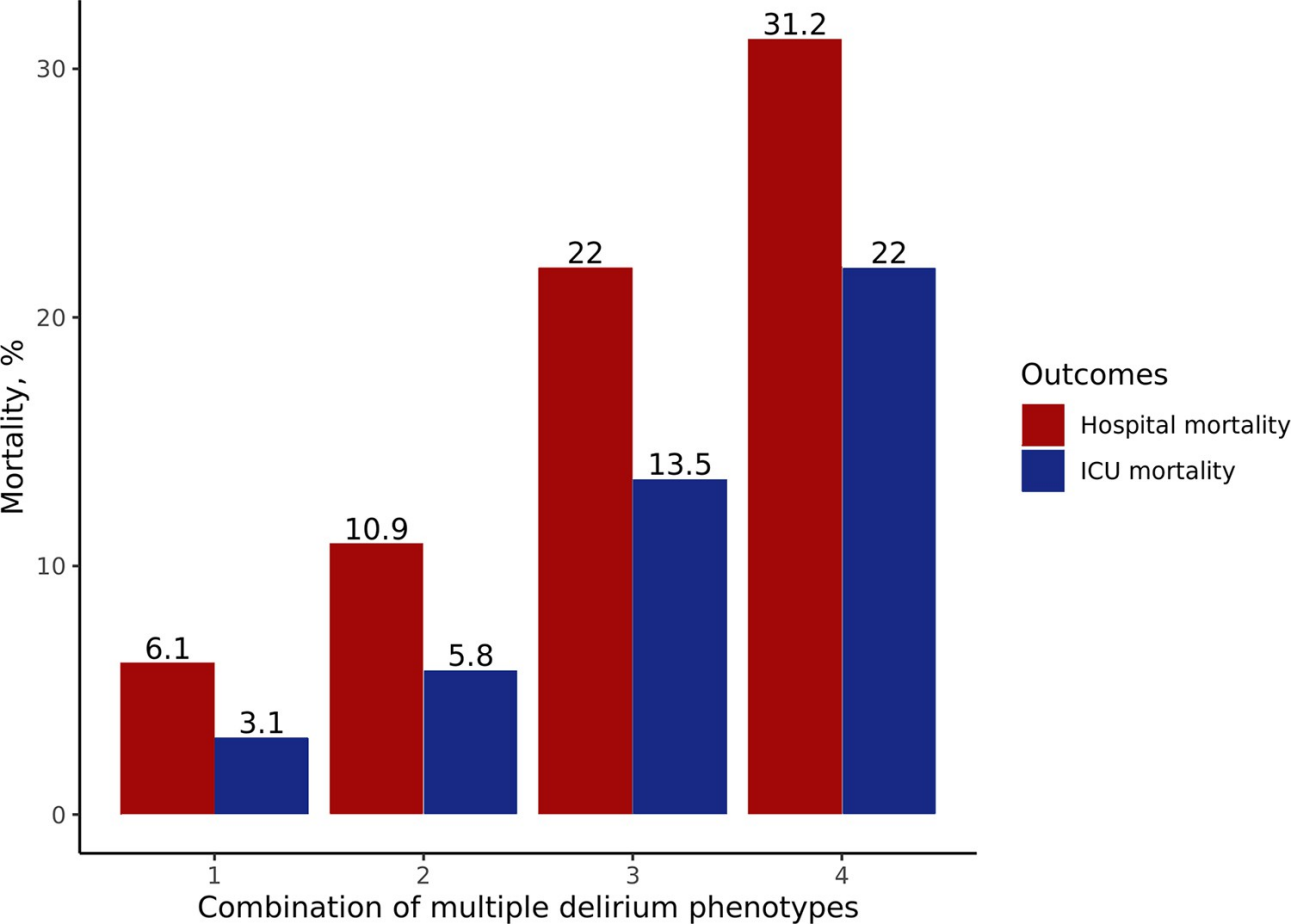
Clinical outcomes depending on the number of combinations of multiple delirium phenotypes. Red bars represent the in-hospital mortality of 599 patients after excluding those who experienced unclassified delirium alone (Pearson’s r: 1.00, p < 0.001). The blue bars indicate the cardiac intensive care unit mortality of 599 patients after excluding those who experienced unclassified delirium alone (Pearson’s r: 1.00, p < 0.001).

The incidence of delirium was 24.4% at the index hospitalization in all CICU patients, and 22.6% within 7 days of CICU admission. Of all patients who developed delirium within 7 days, 393 (62.5%) occurred delirium within one day of CICU admission. Delirium occurred during 2,273 (16.2%) of all 14,073 patient-days during which the mental status could be assessed within 7 days (i.e. days of comatose mentality were excluded). **Fig 1** shows the prevalence of the delirium phenotypes within 7 days. The median duration of each phenotype is shown in **Table 3**. The most common delirium phenotype was septic delirium (17.2%), followed by hypoxic delirium (16.8%). Each delirium phenotype was similar in prevalence. In contrast, unclassified delirium was less frequent than were the other types of delirium. During most delirium days, multiple phenotypes were observed. One delirium phenotype was presented during 336 (14.8%) days, two were present during 666 (29.3%) days, three were present during 646 (28.4%) days, and four were present 240 (10.6%) days. According to the primary diagnosis at the time of CICU admission, delirium was more frequently observed in patients with heart failure, while it was less frequent in patients with acute coronary syndrome (**Table 4**). Sedative-related delirium was relatively more frequent in patients with acute coronary syndrome than it was in patients with heart failure or other reasons for CICU admission. Metabolic delirium was more frequent in patients with acute coronary syndrome or heart failure than were other causes. In patients affected by delirium (within 7 days), both the ICU and hospital mortality increased significantly as the number of delirium phenotypes increased (**Fig 2**, Pearson’s r: 1.00, p < 0.001, both).

**Table 3:** Prevalence and duration of delirium phenotypes within seven days of CICU admission.

	Prevalence among CICU patients (N = 2,783)	Frequency among delirium days (N = 2,273)	Duration among participants affected, day
Any delirium	629 (22.6)	2,273 (100.0)	
Hypoxic	467 (16.8)	1,271 (55.9)	1 (0–3)
Septic	479 (17.2)	1,446 (63.6)	1 (0–1)
Sedative-related	429 (15.4)	1,173 (51.6)	1 (0–3)
Metabolic	338 (12.1)	874 (38.5)	0 (0–2)
Unclassified	133 (4.8)	195 (8.6)	0 (0–0)

Presented values are medians with interquartile ranges in parentheses or numbers with percentages in parentheses.

CICU, cardiac intensive care unit.

**Table 4:** Prevalence of delirium phenotypes according to primary diagnosis.

	Acute coronary syndrome (n = 1,217)	Heart failure (n = 846)	Others (n = 720)	p-value
During CICU stay	209 (17.2)	312 (36.9)	157 (21.8)	< 0.001*^,^**^,^***
Within 7 days	196 (16.1)	289 (34.2)	144 (20.0)	< 0.001*^,^**^,^***
Phenotype				
Hypoxic	148 (75.5)	221 (76.5)	98 (68.1)	0.150
Septic	153 (78.1)	224 (77.5)	102 (70.8)	0.231
Sedative-related	151 (77.0)	190 (65.7)	88 (61.1)	0.004*^,^***
Metabolic	115 (58.7)	163 (56.4)	60 (41.7)	0.004**^,^***
Unclassified	42 (21.4)	51 (17.6)	40 (27.8)	0.052

The presented values are numbers with percentages in parentheses.

*p < 0.05 in the post hoc analysis between the acute coronary syndrome group and the heart failure group.

**p < 0.05 in the post hoc analysis between the heart failure group and others group.

***p < 0.05 in the post hoc analysis between the heart failure group and others group

CICU, cardiac intensive care unit.

According to motor subtypes, the mixed delirium group included more septic and metabolic phenotypes than the other two groups (**Table 5**). The hypoactive delirium group included a less sedative-related phenotype than the other two groups.

**Table 5:** Prevalence of delirium phenotypes according to motor subtypes of delirium.

	Hypoactive (n = 113)	Hyperactive (n = 328)	Mixed type (n = 188)	P
Phenotype				
Hypoxic	79 (69.9)	240 (73.2)	148 (78.7)	0.194
Septic	81 (71.7)	236 (72.0)	162 (86.2)	0.001**^,^***
Sedative-related	55 (48.7)	232 (70.7)	142 (75.5)	<0.001*^,^***
Metabolic	52 (46.0)	176 (53.7)	110 (58.5)	0.109**^,^***
Unclassified	23 (20.4)	82 (25.0)	28 (14.9)	0.025

The presented values are numbers with percentages in parentheses.

*P<0.05 in the post hoc analysis between the hypoactive delirium group and the hyperactive delirium group.

**P<0.05 in the post hoc analysis between the hyperactive delirium group and the mixed delirium group.

***P<0.05 in the post hoc analysis between the hypoactive delirium group and the mixed delirium group.

**Fig 3:**
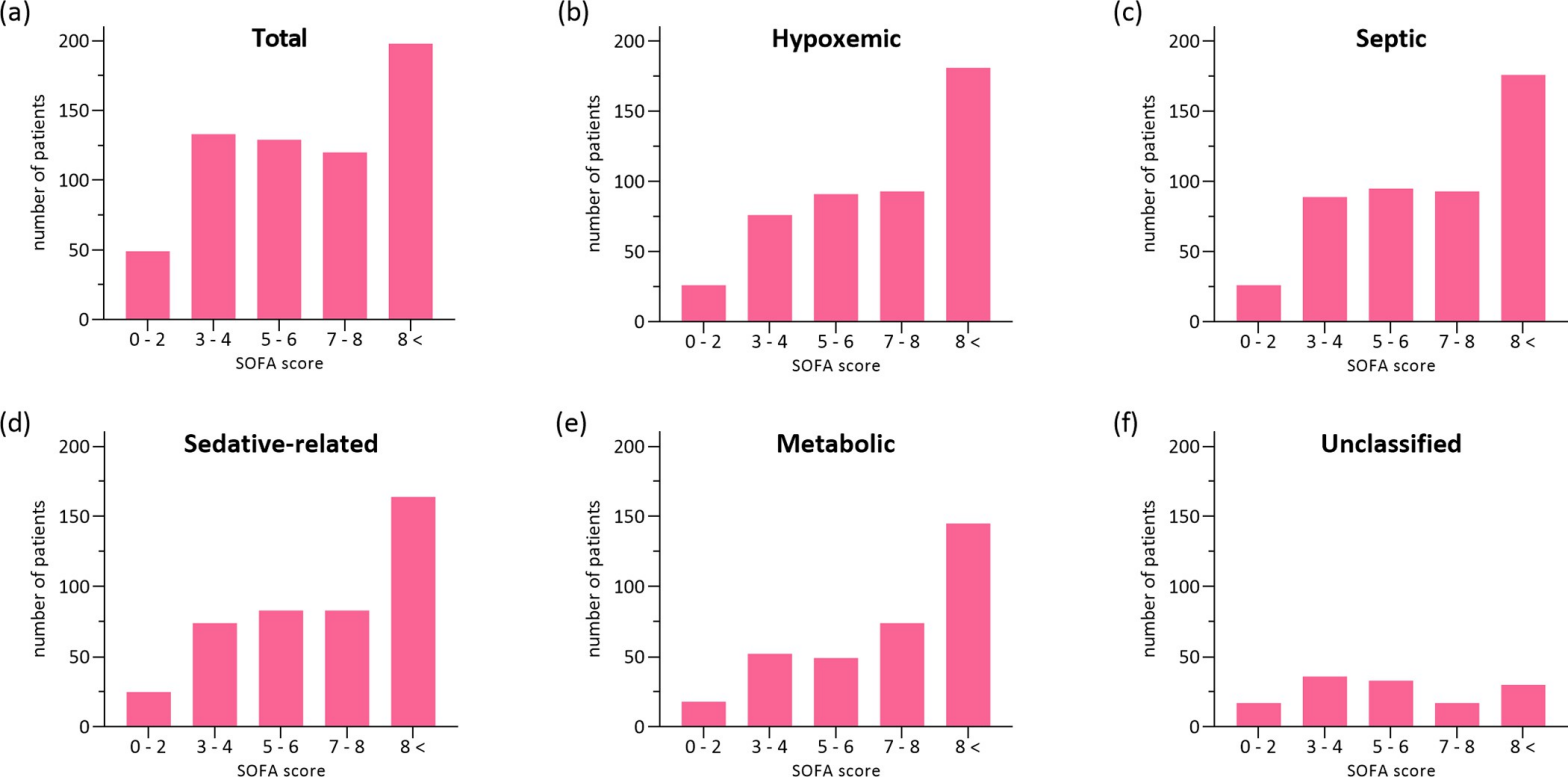
Prevalence of delirium phenotypes according to SOFA score. (A) Total delirium (B) Hypoxemic delirium, (C) Septic delirium, (D) Sedative-related delirium, (E) Metabolic delirium, (F) Unclassified delirium. Number of patients by the SOFA score at CICU admission were presented as bar graphs according to delirium phenotypes.

The prevalence of the delirium phenotype was also compared according to the SOFA score at CICU admission (Fig 3).

## Discussion

We investigated the clinical features of the delirium in patients admitted to the CICU. The delirium incidence was 24.4%. Septic delirium was the most frequently observed delirium phenotype, which was also of longer duration than were the other phenotypes. Of all the patients affected by delirium, the mortality rates increased significantly as the number of concurrent delirium phenotypes increased.

This study investigated the delirium incidence using accurate and frequently measured CAM-ICU assessments. We only included days that could be evaluated on the basis of delirium by excluding any comatose days in patients admitted to the CICU. The delirium incidence was 22.6% and occurred in 16.2% of all patient-days in patients who were admitted over a period of 24 hours within 7 days after admission. Our results show that the incidence of delirium in the CICU is similar to that of the general adult ICUs and surgical ICUs from previous studies [18, 19]. We also found that the incidence of delirium in the CICU is higher than that previously reported in observational studies of other CICUs [7, 17]. We suspect that the reason for this discrepancy in the delirium incidence between our results and those of other CICU studies was caused by clinical characteristics of each study population. Compared with previous studies, our study included all mechanically ventilated CICU patients [17]. We also included more patients on extracorporeal membrane oxygenation than did prior studies (4.5% versus 1%) [7]. In addition, since CAM-ICU was assessed three times per day in patients with a RASS of -3 or higher, our study included more RASS measurements than did prior studies. Finally, we also excluded patients who stayed in the CICU for <24 hours, which may have affected the incidence of delirium in the present study [6].

Long-term cognitive impairment is a known, clinically relevant sequelae of delirium [15, 20]. There was evidence on several occasions to suggest that hypoxia and sepsis affect delirium and subsequently affect patients’ long-term cognitive impairment [21, 22]. There is an increasing number of sepsis patients, with their associated outcomes, in the modern CICU [23, 24]. In our study, the most common delirium phenotype in CICU patients was septic delirium. In contrast, the most common delirium phenotype in a mixed ICU was sedative delirium [15]. This result may reflect modern CICU situations, in which patients are vulnerable to infection because they often receive various percutaneous invasive procedures (such as transcatheter aortic valve replacement, mitral valve repair, and mechanical circulatory support).

Numerous CICU patients may be affected by hypoxic circumstances such as pulmonary congestion or cardiogenic shock. Particularly, in patients with acute heart failure, low cardiac output could induce cerebral hypo-perfusion and reduced plasma clearance of medications which could then influence delirium [25, 26]. In addition, several studies conducted to patients with cardiogenic shock also reported an altered mental state as a symptom of clinical hypo-perfusion. Actually, The IABP-SHOCK II (Intraaortic Balloon Support for Myocardial Infarction With Cardiogenic shock) randomized trial reported that up to 75% exhibited an altered mental state which was a sign of impaired organ perfusion [27]. The CardShock registry also portrayed a similar result; up to 68% showed signs of mental confusion [28]. Furthermore, these patients often require mechanical circulatory support and spend several days in CICU until they reach a more stable condition. CICU patients may be more vulnerable to frailty because two-thirds of all patients with cardiovascular disease are over 60 years of age [29]. Older patients are less tolerant of hypoxic-ischemic brain injury than younger patients and may be more susceptible to hypoxia [30]. Therefore, there needs to be more focus on this area of concern relating to delirium in elderly patients with heart failure and cardiogenic shock is required.

### Study limitations

Although this study provides additional information on delirium in the CICU with a relatively large sample size, our study also has certain limitations that should be acknowledged. Firstly, the study was limited by its inherent retrospective and observational nature. Therefore, detailed information including medication before admission and frailty were not included. However, we tried to collect sufficient data from a clinical data warehouse integrated into a retrospective CICU registry which data collected by a fully trained study coordinator. However, there was still a potential risk of omitting data and/or a potential effect of unrecorded medical events. Secondly, our study was conducted at a single level 1 care center. Therefore, our results could be subject to selection bias and may not be generalizable to other populations. Thirdly, this study did not evaluate the long-term cognitive outcomes in our CICU patient cohort. Therefore, it is necessary to tailor a prospective cohort study to evaluate the severity of any long-term cognitive impairment according to the clinical delirium phenotype.

## Conclusion

Delirium occurred in about a quarter of patients admitted to the modern CICU. Although the most common delirium phenotype was septic delirium, multiple phenotypes were observed during most delirium days. Both ICU and hospital mortality significantly increased according to the increasing combined number of delirium phenotypes. More efforts are needed to reduce the clinical risk factors of delirium, and to prevent it to improve clinical outcomes in the CICU.

## Supporting information

S1 Table Definition of delirium phenotypes. (DOCX)

## Abbreviations

CICU: cardiac intensive care unit
ICU: intensive care unit
CAM-ICU: Confusion Assessment Method for the intensive care unit
RASS: Richmond Agitation-Sedation Scale
SOFA: Sequential Organ Failure Assessment
