# Supplementary material for: Clinical phenotypes of delirium in patients admitted to the cardiac intensive care unit
Source: PLoS One. 2022 Sep 2;17(9):e0273965. doi: 10.1371/journal.pone.0273965 (PMC9439246; doi:10.1371/journal.pone.0273965)
Supplement: S1 Table — (DOCX) [file pone.0273965.s001.docx]

**Table S1** Definition of delirium phenotypes

| **Phenotype** | **Definition** |
| --- | --- |
| Hypoxic delirium | Hypoxemia^*^ or  shock^**^ |
| Septic delirium | Known or suspected infection and  2+systemic inflammatory response syndrome criteria^***^ |
| Sedative-associated delirium | Receipt of benzodiazepine or  propofol or  opioid or  dexmedetomidine |
| Metabolic delirium | Blood urea nitrogen > 80 mg/dL or  glucose < 45 mg/dL or  International normalized ratio > 2.5 and [aspartate transaminase or alanine transaminase] >200 or  Sodium <120 mmol/L or  Sodium >160 mmol/L |
| Unclassified delirium | None of above |

^*^Two or more 15-minute intervals during which lowest blood oxygen saturation level was <90%

^**^Lactate >4.4 mmol/L or two or more 15 minutes intervals during which the lowest mean arterial pressure was <65 mm Hg

^***^Temperature >38℃ or <36℃, heart rate >90 beats per minute, respiratory rate higher than 20 breaths per minute or PaCO_2_ <32 mm Hg, or leucocyte >12,000/mm^2^ or <4,000/mm^2^
